# The Cytochrome P450 Engineering Database: integration of biochemical properties

**DOI:** 10.1186/1471-2091-10-27

**Published:** 2009-11-12

**Authors:** Demet Sirim, Florian Wagner, Andrey Lisitsa, Jürgen Pleiss

**Affiliations:** 1 Institute of Technical Biochemistry, University of Stuttgart, Allmandring 31, 70569 Stuttgart, Germany; 2 Institute of Biomedical Chemistry of Russian Academy of Medical Sciences, 10, Pogodinskaya ul., 119832 Moscow, Russia

## Abstract

**Background:**

Cytochrome P450 monooxygenases (CYPs) form a vast and diverse enzyme class of particular interest in drug development and a high biotechnological potential. Although very diverse in sequence, they share a common structural fold. For the comprehensive and systematic comparison of protein sequences and structures the Cytochrome P450 Engineering Database (CYPED) was established. It was built up based on an extensible data model that enables its functions readily enhanced.

**Description:**

The new version of the CYPED contains information on sequences and structures of 8613 and 47 proteins, respectively, which strictly follow Nelson's classification rules for homologous families and superfamilies. To gain biochemical information on substrates and inhibitors, the CYPED was linked to the Cytochrome P450 Knowledgebase (CPK). To overcome differences in the data model and inconsistencies in the content of CYPED and CPK, a metric was established based on sequence similarity to link protein sequences as primary keys. In addition, the annotation of structurally and functionally relevant residues was extended by a reliable prediction of conserved secondary structure elements and by information on the effect of single nucleotide polymorphisms.

**Conclusion:**

The online accessible version of the CYPED at http://www.cyped.uni-stuttgart.de provides a valuable tool for the analysis of sequences, structures and their relationships to biochemical properties.

## Background

Cytochrome P450 monooxygenases (CYPs) constitute one of the largest superfamilies of enzymes, spread widely among species of microorganisms, plants, animals, and humans. Since they catalyze the oxidation of a wide range of endogenous compounds in biosynthetic and biodegradation pathways, as well as xenobiotics such as drugs and environmental contaminants [1], an understanding of the substrate specificities of CYPs is crucial for successful drug development and biotechnological applications [2]. CYPs require interaction with a reductase, either as separate protein or as fusion protein [3].

We established the CYPED [4] as a tool for a comprehensive and systematic comparison of CYP sequences and structures, which share only a very low percentage of sequence identity between the superfamilies [5]. For this purpose seed sequences have been extracted from the Cytochrome P450 Homepage [6], incorporated in our in-house data warehouse system DWARF [7], updated by a BLAST [8] search and assigned to homologous families and superfamilies according to the recommended classification scheme [9]. Since the publication of the CYPED, it was applied to identify selectivity and specificity determining residues [10], to adjust CYP families in the Fungal Cytochrome P450 Database [11] and served as a template to design other protein family databases [12,13]. The amount of available CYP sequences and structures almost doubled. Therefore, besides integrating new sequences and structures, we extended the CYPED by biochemical properties, and by adding new functionalities:

• Information on P450-catalyzed reactions, substrate preferences, induction and inhibition is made available by the CPK [14]. Since the protein identifiers of the two databases CYPED and CPK could not be related un-ambiguously, an algorithm which uses a metric based on sequence similarities was developed to link protein entries.

• Information on single-nucleotide polymorphism in human CYP sequences was extracted from the CYPallele homepage [15].

• CYPs share highly conserved secondary structure elements [16]. Therefore it was possible to reliably predict these elements from sequence and annotate them in the CYPED.

## Construction and content

### Database establishment

Homologous families and superfamilies were named according to the Cytochrome P450 Homepage [6] and filled with consistently named CYP sequences from the first version of the CYPED. Thus, seed sequences for almost 400 superfamilies could be identified. Positions 1-499 were annotated as P450-domain to avoid loading reductases into the CYPED while updating fusion enzymes. For each seed sequence a BLAST search [8] was performed in the non-redundant sequence database at NCBI http://www.ncbi.nlm.nih.gov with an E-value of 10^-100^. For each hit, information on sequence, position specific annotations, functional descriptions, and the source organism was extracted and loaded by an automated retrieval system into an in-house developed relational database system [7]. In 28% of the entries the correct CYP name according to Nelson's classification [9] was provided in the NCBI database entry. In 1% the name was in contrast to sequence similarity, and therefore the protein was re-assigned. 1% of the proteins had a name which does not exist according to Nelson's classification, and therefore were assigned to the most similar existing family. Entries which were lacking information on the CYP name were assigned to a family by sequence similarity. Thus 64% of the proteins could be assigned which have not been classified yet. All sequences which were assigned only based on sequence similarity were labelled by "homologous protein of family X (BY SIMILARITY)". 218 proteins without CYP name information and no sequence similarity to existing families, as well as 279 protein fragments were discarded. Following this procedure the entries of the CYPED are consistent with the recommendations of the nomenclature committee.

Sequence entries that originate from the same organism and share a sequence identity of at least 98% are assigned to a single protein entry. For proteins with multiple sequence entries, the longest sequence was defined as reference sequence of the respective protein. Protein structures were downloaded from the Protein Data Bank (PDB) [17] and stored as structural monomers. Secondary structure information was calculated using DSSP [18]. Information on structurally or functionally relevant residues was extracted from the GenBank and annotated in the CYPED.

### New features and functionalities

The current version of the CYPED also provides a feature page for each protein entry where the sequence is displayed and annotations are highlighted. A newly developed dynamic web interface directly incorporates changes in the database.

**Figure 1:**
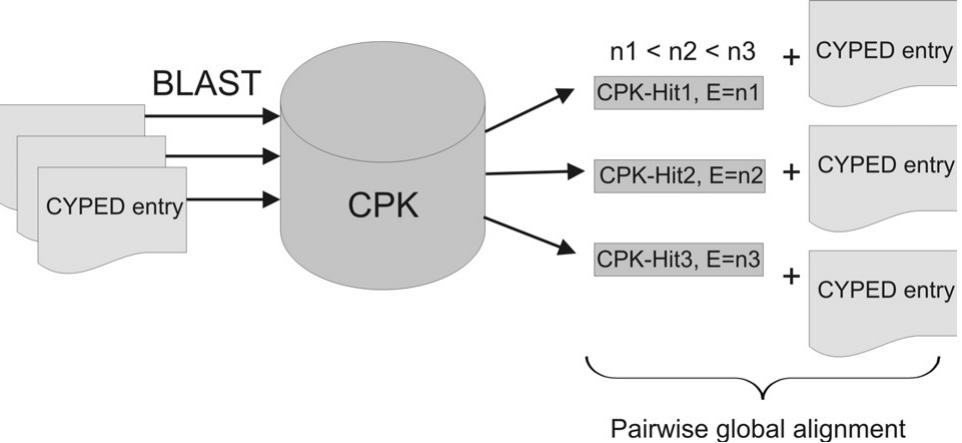
**CYPED - CPK integration pipeline**. Identification and assignment algorithm of the CYPED proteins and the corresponding CPK entries. The steps of the algorithm involve a BLAST search of each of the CYPED entries against the CPK, a ranking of the hits by E-value and a final pairwise alignment of the original CYPED entry with the corresponding CPK-hits to obtain the percentage identity.

The integration of protein databases is based on a common, unique key. A database-independent attribute of a protein which can be applied like a primary key is the protein sequence itself. While a primary key has to be specific, sequences can slightly vary although they might belong to the same protein entry. To overcome this problem, an algorithm was implemented (figure 1) which allows the direct use of the sequences as primary keys without the requirement of being completely identical which was termed a metric primary key. For each CYPED entry, a BLAST search against the CPK database was performed. The BLAST hits were ranked by E-value and a global pairwise alignment was performed [19]. The CPK entries with a sequence identity of more than 90% are displayed on the protein feature page, linking to the corresponding CYPED sequence to the respective entries in the CPK. Thus, the sequences can be applied as common attribute of protein entries and serve as primary keys.

For all sequence entries the conserved secondary structures were predicted by a structure-based HMM-profile which was embedded in an automated annotation program, stored as annotations in the DWARF-system and are displayed on the protein feature pages and within the multisequence alignments.

Information on human CYP alleles was extracted from the "Home Page of the Human Cytochrome P450 (CYP) Allele Nomenclature Committee" [15] and stored in tables designated for this purpose in the database. The mutations and their effect, whether the enzymes lack of activity or gained increased activity, are listed on the protein feature page.

### Contents

The CYPED contains 11193 sequence entries for 8613 protein entries. The proteins have been assigned to 249 superfamilies and 619 homologous families. Structure information for 47 different proteins which originate from 36 different homologous families was extracted from 228 PDB entries.

In total, 3575 CYPED proteins matched the respective CPK entries with a sequence identity of more than 90%. These matches provided the links to 3257 different compounds (1699 substrates, 723 inducers and 1227 inhibitors). This information has been extracted from more than 10000 research papers cited in PubMed [14].

For each family, a multisequence alignment and a phylogenetic tree were generated by CLUSTALW [20]. The annotated version is colour-coded and highlights functionally relevant sites and the predicted secondary structure. For each alignment, the degree of conservation of each column is indicated on the bottom as a coloured chart as calculated by PLOTCON [21]. For each homologous family and superfamily, family specific HMM profiles http://hmmer.janelia.org/ are supplied.

## Utility and discussion

The online version of the CYPED can be browsed by families, source organisms, or structures. Pre-calculated multisequence alignments and structural monomers are displayed and can be downloaded. Phylogenetic trees are visualized by the program PHYLODENDRON http://iubio.bio.indiana.edu/treeapp/. Sequences in the alignments and trees can either be displayed with their accession codes or their systematic names and source organisms as identifiers and they are linked to the respective GenBank entry. For each superfamily and homologous family, family-specific HMM-profiles are provided, which can be applied for the classification and the identification of new CYP sequences. Besides, the website provides a local BLAST interface where a homology search can be performed against the CYPED.

For the 3575 protein entries in the CYPED that match CPK entries, the interface to the CPK provides biochemical information on substrates, inhibitors, and inducers. In the CPK, effectors of CYPs are separated into drugs and non-drugs. Special tags show the cases, when the same compound was reported as substrate and inducer, or when experimental results negated the CYP activity towards a certain chemical species. Through the hyperlink the user is also provided with the information on the PubMed references reporting the relationship between a CYP isoform and low-molecular effector.

## Conclusion

The Cytochrome P450 Engineering Database (CYPED) provides a collection of tools for classification and analysis of the vast and diverse family of cytochrome P450 monooxygenases. To gain a better understanding in biochemical properties and sequence-structure-function relationships, the features of the CYPED were extended by integrating biochemical information from the CPK. Thus, the CYPED has become a valuable tool to navigate in sequence space and to analyze sequence-structure-function relationships.

## Availability and requirements

All sequences, multisequence alignments, phylogenetic trees, and HMM profiles of the Cytochrome P450 Engineering Database (CYPED) are accessible via a web interface at http://www.cyped.uni-stuttgart.de. Additionally, all data is supplied for download.

## List of abbreviations

CYP: Cytochrome P450 monooxygenase; CYPED: Cytochrome P450 Engineering Database; CPK: Cytochrome P450 Knowledgebase; DWARF: Data Warehouse System for Analyzing Protein Families; BLAST: Basic Local Alignment Search Tool; HMM: Hidden Markov model; DSSP: Define Secondary Structure of Proteins.

## Authors' contributions

DS established the database and wrote the manuscript. FW designed and implemented the integration algorithm and generated the web interface. AL provided the CPK data and contributed to the data integration and to the manuscript. JP supervised the project and finalized the manuscript. All the authors have read and approved the final version of the manuscript.
